# Breast density and polymorphisms in genes coding for CYP1A2 and COMT: the Multiethnic Cohort

**DOI:** 10.1186/1471-2407-7-30

**Published:** 2007-02-12

**Authors:** Yumie Takata, Gertraud Maskarinec, Loïc Le Marchand

**Affiliations:** 1 Cancer Research Center of Hawaii, 1236 Lauhala Street, Honolulu, HI, USA

## Abstract

**Background:**

Mammographic density is a strong predictor of breast cancer risk and is increased by hormone replacement therapy (HRT). Some associations with genetic polymorphisms in enzymes involved in estrogen metabolism have been described. This cross-sectional analysis examined the relation between mammographic density and the CYP1A2*1F and *COMT Val*^58 ^*Met *polymorphisms among 332 breast cancer cases and 254 controls in the Hawaii component of the Multiethnic Cohort.

**Methods:**

Mammographic density, before diagnosis in cases, was quantified by using a validated computer-assisted method. Blood samples were genotyped by standard PCR/RFLP methods. Adjusted mean percent density was calculated by genotype using mixed models with the unstructured covariance option.

**Results:**

A positive association between the C allele in the CYP1A2*1F gene and percent density, but not the dense area, was suggested (*p *= 0.11). The relation was limited to controls (*p *= 0.045), postmenopausal women not using HRT (*p *= 0.08), and normal weight subjects (*p *= 0.046). We did not observe any relation between the *COMT Val*^58 ^*Met *polymorphism and breast density.

**Conclusion:**

The lack of an association between the *CYP1A2 *genotype and the size of the dense areas suggests an effect on the non-dense, i.e., fatty breast tissue. The discrepancies among studies may be due to differential susceptibility; changes in enzyme activity as a result of the CYP1A2*1F polymorphism may influence breast tissue differently depending on hormonal status. Larger studies with the ability to look at interactions would be useful to elucidate the influence of genetic variation in *CYP1A2 *and *COMT *on the risk of developing breast cancer.

## Background

Mammographic density has been shown to be independently associated with breast cancer risk [1]. Although a link between mammographic density and circulating estrogen levels was only reported in one out of four reports [2-5], postmenopausal hormone replacement therapy (HRT) appears to increase breast density in observational and experimental studies [6,7]. In addition, associations with genetic polymorphisms in enzymes involved in estrogen metabolism have been explored [8-14]. A previous study in Hawaii with predominately premenopausal women showed lower mammographic densities for women with the C allele in the CYP1A2*1F gene and the Met allele in the *COMT *gene [10]. The CC genotype for CYP1A2*1F was also significantly associated with lower serum estradiol levels during the luteal phase [15]. The particular polymorphisms are thought to lead to lower enzyme activity and they had been associated with breast cancer risk in some reports [16,17]. Also, higher CYP1A2 activity as assessed by urinary caffeine metabolites was associated with higher mammographic densities among postmenopausal women with high malondialdehyde (MDA) levels, an indicator of lipid peroxidation [11]. In a case-control study nested within the Multiethnic Cohort (MEC), postmenopausal women with at least one C allele in the CYP1A2*1F gene had a lower risk of breast cancer than women with the common alleles [18], but a case-control study in Shanghai with women aged 25–64 years did not observe this association [19]. As for the Met allele in the *COMT *gene, higher breast density was found for postmenopausal HRT users in one report [8], lower breast density in women not using HRT in another study [9], lower breast density in pre- but not postmenopausal women [12], higher increase in breast density in response to estrogens during an intervention [14], and no association among postmenopausal women [13]. To clarify the conflicting results related to these two polymorphisms, we linked the aforementioned case-control study [18] with a mammographic case-control study also nested within the MEC that had collected multiple mammograms over time [20] and examined the association between breast density and the CYP1A2*1F and the *COMT Val*^58 ^*Met *polymorphisms among the overlapping subjects.

## Methods

### Study population

The subjects for this analysis were originally recruited for two separate case-control studies [18,20] nested within the MEC study [21]. Both studies were approved by the Human Subject Committee at the University of Hawaii. All participants completed an informed consent form. Controls were randomly selected from the cohort and frequency matched by age and ethnicity for both studies. The genetic polymorphism case-control study included 1,339 breast cancer cases and 1,370 controls from Hawaii and Los Angeles [18]. Incident breast cancer cases since 1995 were identified through the rapid reporting system of the Hawaii Tumor Registry and the Los Angeles County Cancer Surveillance Program. Cases and controls who agreed to participate in the study (74% for cases and 66% for controls) donated a blood sample. For the mammographic density case-control study [20], all incident cases diagnosed with breast cancer in Hawaii between cohort entry and December 2000 were eligible. Of them, 51% agreed to be in the study and for 44% of the eligible subjects a mammogram was located. The final sample size was 607 cases and 667 controls. After linking the breast density study with the genetic polymorphism study, 575 subjects had mammographic and genetic information available.

### Mammographic analysis

To assess breast density, the cranio-caudal views for both sides of several mammographic examinations were retrieved [20]. All mammograms for cases were performed before treatment for breast cancer was initiated and only five mammograms were taken at the time of diagnosis. The films were scanned after removing personal identifiers and quantified with a computer-assisted method by one reader [22]. The total and the dense areas of the breast were estimated, percent density was calculated as the ratio of the dense to the total area of the breast, and the values for the right and the left breast were averaged. For a subset of mammograms read in duplicate, the intraclass correlation coefficient was 0.96 for the size of the dense areas and 0.996 for the total breast area, resulting in a value of 0.974 for percent density.

### Genetic analysis

DNA was extracted from white blood cells and genotyped by PCR/RFLP methods for the *COMT Val*^58 ^*Met *and CYP1A2*1F (A-154C) polymorphisms as described previously [18].

### Statistical analysis

All statistical analyses were performed using SAS version 9 (Cary, NC) [23]. In order to estimate adjusted mean percent density, mixed models with the unstructured covariance option were applied while adjusting for age at mammogram, BMI, case-control status, HRT use and type (estrogen vs. combined therapy), ethnicity, parity, age at menarche, age at first live birth, family history of breast cancer, and menopausal status, where appropriate. A square root transformation was applied to percent density because the variables were not normally distributed. We repeated some models using the log-transformed size of the dense areas as the dependent variable. Mixed models were used to account for the multiple density measures per person. The availability of repeated density assessments increases the statistical power of the analysis because it decreases the intraindividual variation in density measures [24]. Age, menopausal status, and HRT use were included as time dependent variables; all other variables were only assessed once. Genotypes were coded according to the number of variant alleles as none, one, and two to test for a codominant model; coding for a recessive and a dominant model was also performed. Adjusted mean percent densities were calculated according to the number of variant alleles among all women and stratified by case status, ethnicity, menopausal status, and HRT use that were hypothesized a priori as possible modifiers. We also tested for interaction effects of genotype with these variables. Logistic regression was used to estimate the risk of breast cancer related to the two genetic polymorphisms.

## Results

Due to the study design, nearly half of the 575 subjects were breast cancer cases (Table 1). On average, three mammograms were included per woman, but the number of scanned films was higher for cases than for controls. At the time of the first mammogram, the mean age was 57.1 ± 9.3 years and 67% of subjects were postmenopausal. By the time of the last mammogram, on average 4.7 years later, 80% of women were postmenopausal. The median time between first mammogram and breast cancer diagnosis was 6.6 years. Japanese women constituted the largest ethnic group, followed by Caucasians and Native Hawaiians. Approximately two-thirds of women had used HRT at the time of at least one mammogram, but overall 44% of mammograms were performed without HRT use. A higher proportion of controls than cases reported ERT use, whereas combined HRT use was more common among cases. As expected, mean percent density was significantly higher among cases than controls; 36.7% for cases vs. 30.6% for controls.

Adjusted percent densities were significantly higher with an increasing number of variant alleles in the CYP1A2*1F polymorphism among all women; the difference between the AA and CC genotypes was 5.1% (*p *for trend = 0.11) (Table 2). The respective *p*-values for the recessive and the dominant model were 0.23 and 0.18 with density differences of 4.1% and 2.2% between groups (data not shown). Although we detected no significant interaction effects between the CYP1A2*1F polymorphism and case status, menopausal status, and BMI (*p *= 0.13, 0.38, and 0.37, respectively), we noted a number of differences after stratification by these variables. The association between the CYP1A2*1F allele and percent density was stronger among controls than cases. The difference between subjects with AA and CC genotype was 8.9% (*p *= 0.045), while there was no significant difference in breast density by genotype among cases (*p *= 0.86). Given the small number of mammograms among premenopausal women, the 9.6% difference between the two homozygous groups was not significant (*p *= 0.38). Among postmenopausal women, the 9.1% difference for mammograms taken without HRT use was close to significance (*p *= 0.08), whereas the difference among HRT users was only 2.5% (*p *= 0.48). Similarly, the positive association was limited to women with a BMI of less than 25 kg/m^2 ^(9.4%, *p *= 0.046), whereas percent density did not vary by genotype among overweight and obese women. Stratification by ethnicity showed non-significant higher mean densities for Japanese CC carriers but no relation among Caucasians. The mean densities for the Japanese AA, AC, and CC carriers were 32.9%, 31.7%, and 39.4% (*p *= 0.44); the respective values for Caucasians were 26.0%, 27.9%, and 25.1% (*p *= 0.60).

Genotypes for *COMT Val*^58 ^*Met *were not associated with percent density in the entire population or in any subgroup (Table 2). The density difference between homozygous genotypes was less than 1% in the entire population. The respective *p*-values for the recessive and the dominant model were 0.83 and 0.70. No interaction effect of *COMT Val*^58 ^*Met *with case status, menopausal status, or BMI was suggested (*p *= 0.71, 0.90, and 0.20, respectively).

When we used the size of the dense areas as dependent variable, we found no difference in mean densities by genotype for *CYP1A2 *and *COMT *(*p *= 0.85 and 0.98).

Compared to women with the AA genotype, the risk estimates for breast cancer associated with one or two C alleles in the *CYP1A2 *gene were 0.86 (95% CI = 0.60–1.23) and 0.62 (95% CI = 0.30–1.30), respectively. For the *COMT Val*^58 ^*Met *polymorphism, the OR was 0.93 (95% CI = 0.64–1.35) for one Met allele and 0.61 (95% CI = 0.37–1.02) for two Met alleles as compared to the women with two Val alleles.

## Discussion

In this analysis of longitudinal mammographic data, we observed a weak association between percent density and the number of C alleles in the CYP1A2*1F gene, but the relation was limited to controls, postmenopausal women not using HRT, and normal weight women. The fact that the size of the dense areas was not associated with the *CYP1A2 *genotype suggests that the relation may be due to an effect on the non-dense, i.e., the fatty breast tissue. We did not observe any relation between percent density and the Met polymorphism in the *COMT *gene.

In comparison, a Canadian study reported mean percent densities of 14% for the lowest and 31% for the highest quartile of CYP1A2 activity (*p *for trend = 0.01) among postmenopausal women not using HRT [11]. The current findings are inconsistent with studies conducted in Hawaii that described lower percent densities [10] and estrogen levels [15] in premenopausal carriers of the C allele, as well as a lower breast cancer risk among postmenopausal women with at least one C allele for CYP1A2*1F [18]. However, the estimated risks of 0.86 (95% CI = 0.60–1.23) for heterozygotes and 0.62 (95% CI = 0.30–1.30) for women with the CC genotype in the current study are very similar to the risk estimates in the larger case-control study (OR = 1.0, 0.9 and 0.7 for the AA, AC, and CC genotypes; *p *for trend = 0.03) that also included Latina and African-American women in Los Angeles. The findings with regard to the *COMT Val*^58 ^*Met *polymorphism are also inconsistent. Studies in Hawaii [10] and in Canada [12] suggested lower breast densities for premenopausal carriers of the Met allele. In contrast, one recent study found no relation among postmenopausal women [13] and two reports described associations for subgroups of postmenopausal women with the Met allele: higher breast density for HRT users [8] and lower breast density in women not using HRT [9].

These discrepant results for both genotypes may be due to chance given the limited sample size, but other explanations are also plausible. The small change in hormone levels due to a variant allele may not be observable, especially in light of the weak evidence for a relation between breast density and circulating estrogen levels [2-5]. Our observation that the relation between the *CYP1A2 *genotype and percent density was probably due to an inverse association with the non-dense, i.e., the fatty tissue, may offer a plausible explanation for the discrepancy with the findings on breast cancer risk [18]. As a speculation, we propose that one or two C alleles may be protective against breast cancer because the *CYP1A2 *genotype might influence the amount of adipose tissue in the breast and elsewhere. As apparent from the sex differences in body fat distribution, steroid hormones influence fat tissue, most likely through binding to sex steroid hormone receptors that regulate the production of adipocytokines in adipose tissue [25,26]. Therefore, differences in circulating estrogen metabolite patterns due to altered enzyme activity might modify the amount of adipose tissue in the breast. Alternatively, the causality of the association between genotype and amount of non-dense tissue may be reversed. Because there is evidence that the expression level of CYP1A2 is upregulated by estrogens [27], postmenopausal women with more body fat and higher endogenous estrogen production may have higher CYP1A2 activity. The genetic polymorphism may only affect postmenopausal women with low body fat, low endogenous estrogens, and relatively low CYP1A2 activity, whereas the higher estrogen levels and CYP1A2 activity in women who use HRT [28], will develop breast cancer [29], or are overweight [30] may outweigh any changes in hormone patterns due to the *CYP1A2 *variant alleles. This idea of differential susceptibility would explain why the effects of the *CYP1A2 *genotype were only apparent in certain subgroups and why our previous findings in premenopausal women were so different [10,15]. In addition, a recent experimental study showed that, among all CYP enzymes tested, CYP1A2 has the highest 2-hydroxylation (2-OH) activity on estrone, especially at low concentrations [31]. The positive association between the *CYP1A2 *polymorphism and breast density among controls, women no taking HRT, and normal weight women could be due to the higher levels of 2-OH estrone, a metabolite with low estrogenic activity, among common allele carriers than among women with the variant allele resulting in greater mammographic densities among the latter.

Strengths of this study were our prospective data from a cohort study that minimized recall bias and the retrieval of longitudinal mammographic data. There are a number of serious limitations in our study. For instance, the response rates and proportions of ethnic groups by case status were not optimal [18,20]. Selection bias due to the availability of mammograms may have led to a higher proportion of women at high risk for breast cancer than in the general population. Although we were able to show a strong association between breast cancer diagnosis and breast density in the mammographic density study [20], the scanned mammograms may not have covered the critical time of a woman's life. Unmeasured confounders, such as lipid peroxidation and intake of antioxidants [11,32], may be partially responsible for the contradictory findings. Although *CYP1A2 *activity contributes to estrogen metabolism, polymorphisms in other genes should be considered by themselves and in combination. It is not quite clear yet how much the CYP1A2*1F allele influences actual enzyme activity. When caffeine metabolites were measured, enzyme activity was higher among carriers of the CYP1A2*1F allele in one [33], but not in another report [34]. Given the stronger association of percent density with progesterone than estrogens [3,7], polymorphisms related to progestin metabolism may provide additional insights.

## Conclusion

This study among a subset of MEC members suggests higher percent densities for women carrying at least one C allele in the *CYP1A2 *gene than for those with two A alleles but no association between breast density and the Met allele in the *COMT *gene. It appears that the *CYP1A2 *genotype affects the non-dense area of the breast, but a possible mechanism of action remains to be defined. Given the weak association between percent density and estrogens, the alteration in enzyme activity due to the CYP1A2*1F polymorphism may be too small to influence breast density, in particular among women who are exposed to relatively high hormone levels, such as HRT users, overweight postmenopausal women, and women who will develop breast cancer. Until larger studies with the ability to look at gene-gene and gene-environment interactions are completed, it will remain unclear to what extent the variant alleles for *CYP1A2 *and *COMT *affect a woman's lifetime risk of developing breast cancer.

## Competing interests

The author(s) declare that they have no competing interests.

## Authors' contributions

YT performed the statistical analysis and drafted the manuscript. GM designed the study and helped to perform the statistical analysis and draft the manuscript. LM designed the study, supervised the genetic analysis and critically commented on the drafted manuscript. GM and LM obtained funding for the previous projects. All authors read and approved the final manuscript.

## Pre-publication history

The pre-publication history for this paper can be accessed here:



## Figures and Tables

**Table 1:** Characteristics of the Study Population*

Characteristic	Cases	Controls	All
Number of women	325	250	575
Ethnicity			
Caucasian	112 (34.5)	74 (29.6)	186 (32.4)
Japanese	168 (51.7)	101 (40.4)	269 (46.8)
Native Hawaiian	43 (13.2)	75 (30.0)	118 (20.5)
Number of mammograms	3.4 ± 1.9	2.5 ± 1.2	3.0 ± 1.7
Age at first mammogram (years)	57.4 ± 8.9	56.8 ± 9.9	57.1 ± 9.3
Percent density at first mammogram (%)	36.7 ± 22.8	30.6 ± 23.3	34.0 ± 23.2
Time between first and last mammogram (years)	5.2 ± 3.8	4.2 ± 3.8	4.7 ± 3.8
Postmenopausal at time of first mammogram	233 (71.7)	153 (61.2)	386 (67.1)
Hormone replacement therapy by mammogram			
None	472 (42.5)	290 (47.2)	762 (44.3)
Estrogen only	282 (25.5)	207 (33.7)	489 (28.4)
Estrogen plus progestin	354 (32.0)	117 (19.1)	471 (27.3)
Overweight/obese (BMI ≥ 25 kg/m^2^)	121 (37.2)	113 (45.2)	234 (40.7)
Family history of breast cancer	56 (17.2)	31 (12.4)	87 (15.1)
Age at menarche younger than 13 years	194 (59.7)	136 (54.4)	330 (57.4)
Age at first live birth older than 30 years	29 (8.9)	26 (10.4)	55 (9.6)
Nulliparity	43 (13.2)	31 (12.4)	74 (12.9)
Parity 3+ children	72 (22.2)	53 (21.2)	125 (21.7)

* Mean ± SD are given for continuous measures, counts and percentages for other variables.

**Table 2:** Adjusted Mean Percent Densities (%) by Genotype*

	**CYP1A2**	**N**^#^	**N**^##^	**Density**	**COMT**	**N**^#^	**N**^##^	**Density**
All	AA	863	285	27.8	Val/Val	688	229	28.6
	AC	750	254	29.7	Val/Met	780	257	29.1
	CC	109	36	32.9	Met/Met	254	89	29.4
	*P (trend)*^‡^	-	-	*0.11*	*P (trend)*^‡^	-	-	*0.70*

Controls	AA	315	124	22.4	Val/Val	235	95	23.6
	AC	253	108	25.4	Val/Met	264	108	24.6
	CC	46	18	31.3	Met/Met	115	47	25.1
	*P (trend)*^‡^	-	-	***0.045***	*P (trend)*^‡^	-	-	*0.61*

Cases	AA	548	161	31.1	Val/Val	453	134	31.4
	AC	497	146	31.4	Val/Met	516	149	31.5
	CC	63	18	32.0	Met/Met	139	42	30.3
	*P (trend)*^‡^	-	-	*0.86*	*P (trend)*^‡^	-	-	*0.84*

Premenopausal	AA	154	71	35.2	Val/Val	125	62	35.7
	AC	129	60	36.1	Val/Met	124	55	37.8
	CC	9	4	44.8	Met/Met	43	18	30.8
	*P (trend)*^‡^	-	-	*0.38*	*P (trend)*^‡^	-	-	*0.60*

Postmenopausal	AA	652	189	25.9	Val/Val	523	150	26.5
	AC	566	170	27.4	Val/Met	596	174	27.0
	CC	92	27	31.4	Met/Met	191	62	27.6
	*P (trend)*^‡^	-	-	*0.14*	*P (trend)*^‡^	-	-	*0.67*

Postmenopausal	AA	258	113	20.5	Val/Val	180	90	21.6
No HRT use	AC	186	98	23.1	Val/Met	225	104	21.7
	CC	21	11	29.6	Met/Met	60	28	24.4
	*P (trend)*^‡^	-	-	*0.08*	*P (trend)*^‡^	-	-	*0.57*

Postmenopausal	AA	228	97	29.2	Val/Val	176	81	29.3
HRT use	AC	192	88	30.4	Val/Met	192	79	30.8
	CC	28	14	31.7	Met/Met	80	39	29.2
	*P (trend)*^‡^	-	-	*0.48*	*P (trend)*^‡^	-	-	*0.88*

BMI < 25	AA	504	157	34.2	Val/Val	412	135	35.4
	AC	485	163	37.1	Val/Met	505	154	36.1
	CC	71	21	43.6	Met/Met	143	52	37.8
	*P (trend)*^‡^	-		***0.046***	*P (trend)*^‡^	-	-	*0.53*

BMI >= 25	AA	359	128	19.2	Val/Val	276	94	19.0
	AC	265	81	19.2	Val/Met	275	103	19.4
	CC	38	15	18.6	Met/Met	111	37	18.9
	*P (trend)*^‡^	-		*0.92*	*P (trend)*^‡^	-	-	*0.96*

*Percent densities were computed through mixed models using square-root transformed values while adjusting for age, BMI, case status, HRT use & type, ethnicity, parity, age at menarche, age at first live birth, family history of breast cancer, and menopausal status. Age, HRT use, and menopausal status are time-dependent variables.

# N is the number of mammograms used in the analysis; ## N is the number of subjects.

‡ *P*-values for a trend variable assigned the gene dosage effect using mixed models.

## References

[B1] Boyd NF, Lockwood GA, Byng JW, Tritchler DL, Yaffe MJ (1998). Mammographic densities and breast cancer risk. Cancer Epidemiol Biomarkers Prev.

[B2] Boyd NF, Stone J, Martin LJ, Jong R, Fishell E, Yaffe M, Hammond G, Minkin S (2002). The association of breast mitogens with mammographic densities. Br J Cancer.

[B3] Noh JJ, Maskarinec G, Pagano I, Cheung LW, Stanczyk FZ (2006). Mammographic densities and circulating hormones: a cross-sectional study in premenopausal women. Breast.

[B4] Tamimi RM, Hankinson SE, Colditz GA, Byrne C (2005). Endogenous sex hormone levels and mammographic density among postmenopausal women. Cancer Epidemiol Biomarkers Prev.

[B5] Greendale GA, Palla SL, Ursin G, Laughlin GA, Crandall C, Pike MC, Reboussin BA (2005). The association of endogenous sex steroids and sex steroid binding proteins with mammographic density: results from the Postmenopausal Estrogen/Progestin Interventions Mammographic Density Study. Am J Epidemiol.

[B6] Laya MB, Gallagher JC, Schreiman JS, Larson EB, Watson P, Weinstein L (1995). Effect of postmenopausal hormonal replacement therapy on mammographic density and parenchymal pattern. Radiology.

[B7] Greendale GA, Reboussin BA, Slone S, Wasilauskas C, Pike MC, Ursin G (2003). Postmenopausal hormone therapy and change in mammographic density. J Natl Cancer Inst.

[B8] Haiman CA, Bernstein L, Berg D, Ingles SA, Salane M, Ursin G (2002). Genetic determinants of mammographic density. Breast Cancer Res.

[B9] Haiman CA, Hankinson SE, De Vivo I, Guillemette C, Ishibe N, Hunter DJ, Byrne C (2003). Polymorphisms in steroid hormone pathway genes and mammographic density. Breast Cancer Res Treat.

[B10] Maskarinec G, Lurie G, Williams AE, Le Marchand L (2004). An investigation of mammographic density and gene variants in healthy women. Int J Cancer.

[B11] Hong CC, Tang BK, Rao V, Agarwal S, Martin L, Tritchler D, Yaffe M, Boyd NF (2004). Cytochrome P450 1A2 (CYP1A2) activity, mammographic density, and oxidative stress: a cross-sectional study. Breast Cancer Res.

[B12] Hong CC, Thompson HJ, Jiang C, Hammond GL, Tritchler D, Yaffe M, Boyd NF (2003). Val158Met Polymorphism in catechol-O-methyltransferase gene associated with risk factors for breast cancer. Cancer Epidemiol Biomarkers Prev.

[B13] Warren R, Skinner J, Sala E, Denton E, Dowsett M, Folkerd E, Healey CS, Dunning A, Doody D, Ponder B, Luben RN, Day NE, Easton D (2006). Associations among mammographic density, circulating sex hormones, and polymorphisms in sex hormone metabolism genes in postmenopausal women. Cancer Epidemiol Biomarkers Prev.

[B14] Lord SJ, Mack WJ, Van Den BD, Pike MC, Ingles SA, Haiman CA, Wang W, Parisky YR, Hodis HN, Ursin G (2005). Polymorphisms in genes involved in estrogen and progesterone metabolism and mammographic density changes in women randomized to postmenopausal hormone therapy: results from a pilot study. Breast Cancer Res.

[B15] Lurie G, Maskarinec G, Kaaks R, Stanczyk FZ, Le ML (2005). Association of genetic polymorphisms with serum estrogens measured multiple times during a 2-year period in premenopausal women. Cancer Epidemiol Biomarkers Prev.

[B16] Dunning AM, Healey CS, Pharoah PD, Teare MD, Ponder BA, Easton DF (1999). A systematic review of genetic polymorphisms and breast cancer risk. Cancer Epidemiol Biomarkers Prev.

[B17] Coughlin SS, Piper M (1999). Genetic polymorphisms and risk of breast cancer. Cancer Epidemiol Biomarkers Prev.

[B18] Le Marchand L, Donlon T, Kolonel LN, Henderson BE, Wilkens LR (2005). Estrogen metabolism-related genes and breast cancer risk: the multiethnic cohort study. Cancer Epidemiol Biomarkers Prev.

[B19] Long JR, Egan KM, Dunning L, Shu XO, Cai Q, Cai H, Dai Q, Holtzman J, Gao YT, Zheng W (2006). Population-based case-control study of AhR (aryl hydrocarbon receptor) and CYP1A2 polymorphisms and breast cancer risk. Pharmacogenet Genomics.

[B20] Maskarinec G, Pagano I, Lurie G, Wilkens LR, Kolonel LN (2005). Mammographic Density and Breast Cancer Risk: The Multiethnic Cohort. Am J Epidemiol.

[B21] Kolonel LN, Henderson BE, Hankin JH, Nomura AMY, Wilkens LR, Pike MC, Stram DO, Monroe KR, Earle ME, Nagamine FS (2000). A multiethnic cohort in Hawaii and Los Angeles: baseline characteristics. Am J Epidemiol.

[B22] Byng JW, Boyd NF, Fishell E, Jong RA, Yaffe MJ (1994). The quantitative analysis of mammographic densities. Phys Med Biol.

[B23] Inc. SASI (2004). SAS OnlineDoc 912.

[B24] Singer JD, Willett JB (2003). Applied longitudinal data analysis: Modeling change and event occurrence.

[B25] Mayes JS, Watson GH (2004). Direct effects of sex steroid hormones on adipose tissues and obesity. Obes Rev.

[B26] Laughlin GA, Barrett-Connor E, May S (2006). Sex-specific association of the androgen to oestrogen ratio with adipocytokine levels in older adults: the Rancho Bernardo Study. Clin Endocrinol (Oxf).

[B27] Tsuchiya Y, Nakajima M, Yokoi T (2005). Cytochrome P450-mediated metabolism of estrogens and its regulation in human. Cancer Lett.

[B28] Lee SA, Ross RK, Pike MC (2005). An overview of menopausal oestrogen-progestin hormone therapy and breast cancer risk. Br J Cancer.

[B29] The Endogenous Hormones and Breast Cancer Collaborative Group (2002). Endogenous sex hormones and breast cancer in postmenopausal women: reanalysis of nine prospective studies. J Natl Cancer Inst.

[B30] Key TJ, Appleby PN, Reeves GK, Roddam A, Dorgan JF, Longcope C, Stanczyk FZ, Stephenson HE, Falk RT, Miller R, Schatzkin A, Allen DS, Fentiman IS, Key TJ, Wang DY, Dowsett M, Thomas HV, Hankinson SE, Toniolo P, Akhmedkhanov A, Koenig K, Shore RE, Zeleniuch-Jacquotte A, Berrino F, Muti P, Micheli A, Krogh V, Sieri S, Pala V, Venturelli E, Secreto G, Barrett-Connor E, Laughlin GA, Kabuto M, Akiba S, Stevens RG, Neriishi K, Land CE, Cauley JA, Kuller LH, Cummings SR, Helzlsouer KJ, Alberg AJ, Bush TL, Comstock GW, Gordon GB, Miller SR, Longcope C (2003). Body mass index, serum sex hormones, and breast cancer risk in postmenopausal women. J Natl Cancer Inst.

[B31] Cribb AE, Knight MJ, Dryer D, Guernsey J, Hender K, Tesch M, Saleh TM (2006). Role of polymorphic human cytochrome P450 enzymes in estrone oxidation. Cancer Epidemiol Biomarkers Prev.

[B32] Wiseman A (2005). Oestrogen-receptors (ER) are likely to be promiscuous: wider role for oestrogens and mimics. Med Hypotheses.

[B33] Sachse C, Brockmoller J, Bauer S, Roots I (1999). Functional significance of a C-->A polymorphism in intron 1 of the cytochrome P450 CYP1A2 gene tested with caffeine. Br J Clin Pharmacol.

[B34] Takata K, Saruwatari J, Nakada N, Nakagawa M, Fukuda K, Tanaka F, Takenaka S, Mihara S, Marubayashi T, Nakagawa K (2006). Phenotype-genotype analysis of CYP1A2 in Japanese patients receiving oral theophylline therapy. Eur J Clin Pharmacol.

